# Mitochondrial genome and phylogenetic analysis of Gaojiao chicken (*Gallus gallus*)

**DOI:** 10.1080/23802359.2020.1765707

**Published:** 2020-05-20

**Authors:** Lili Liu, Kui Ren, Yuanchang Jin, Huimin Zeng

**Affiliations:** a College of life science, Hunan University of Science and Technology, Xiangtan, China; b Hunan Key Laboratory of Economic Crops Genetic Improvement and Integrated Utilization, Hunan University of Science and Technology, Xiangtan, China

**Keywords:** Mitogenome, Gaojiao chicken, phylogenetic analysis

## Abstract

In this study, the complete mitochondrial genome of Gaojiao chicken (*Gallus gallus*) was sequenced using samples collected from Puding county of Guizhou province, China. The mitogenome of Gaojiao chicken is 16,786 bp in length, and it contained 22 transfer RNA (tRNA) genes, 2 ribosomal RNA (rRNA) genes, 13 protein-coding genes (PCGs), and a control region (D-loop), the overall base nucleotide compositions encoded are 30.27% A, 32.52% C, 13.49% G, and 23.72% T. The phylogenetic tree was constructed using neighbor-joining (N-J) method, which indicated that the Gaojiao chicken has the close relationship with Nandan chicken, Daweishan chicken, Wuding chicken, and Guangxi three-buff chicken.

Gaojiao chicken is a local breed in Guizhou Province of China. It has long legs and wings, and the tibia is about 3–4 cm longer than other breeds. Gaojiao chicken is very popular because of its better meat quality and delicious taste. However, there are some disadvantages for Gaojiao chicken, the growth and development of individual is relatively slow, lower egg production and reproduction rate, and lower feed reward, so the number of raised-chickens decreased significantly, and they are almost on the survival-risk breed (Yang et al., 2015). The sampling location used in this study was Puding County (E105°15′–105°49′, N25°34′–26°05′) in the center of Guizhou province, China. Total DNA was extracted from the specimens (Voucher No. GJC160805) were stored at −70 °C in the Laboratory of Molecular Biology, College of life science, Hunan University of Science and Technology. We reported the sequence of the complete mitogenome of Gaojiao chicken for the first time, and the complete mitochondrial DNA data from this study have submitted to GenBank and got the accession number (MT177340). According to mitochondrial genome of Silky chicken (AB086102), the 22 pairs of primers for amplifying the complete mitochondrial DNA was designed, and the polymerase chain reaction (PCR) products of the Gel electrophoresis were purified by Gel Advanced™ Gel Extraction (Rich Biotech, China) and sequenced by BioSune Biotech (Shanghai, China). The characters of base composition and distribution were analyzed using tRNA Scan-SE1.21 and DOGMA software (Liu et al. 2016, 2018). The mitochondrial DNA sequence was analyzed using the DNAStar7.1 software (Madison, WI). The phylogenetic tree was constructed using MEGA7.0 and NJ Algorithm software based on D-loop sequence of Gaojiao chicken and other breed chickens.

The results revealed that the total length of mitochondrial sequence of Gaojiao chicken is 16,786 bp, with the base composition of 30.27% for A, 23.72% for T, 32.52% for C, 13.49% for G, with high a A + T content of 53.99%. The length of non-coding region is 1232 bp, which accounts for 7.34% of the total length, and as the D-loop region. The length of coding region is 15,554 bp and it contained 37 coding genes (22 tRNA genes, 2 rRNA genes, and 13 protein-coding genes). One protein-coding gene (*ND6*) and 8 tRNA genes (*tRNA^Cys^*, *tRNA^Tyr^*, *tRNA^Ser^*, *tRNA^Pro^*, *tRNA^Glu^*, *tRNA^Gln^*, *tRNA^Ala^*, and *tRNA^Asn^*) were encoded on the light (L) strand. However, the other 12 protein-coding genes, 14 tRNA and 2 rRNA genes were encoded on the heavy (H) strand. The initiation codon of proteins genes was ATG except for COX1 being GTG. There are four types of termination codon for proteins genes, including TAA, TAG, AGG, and an incomplete termination codon “T–”, which is the 5′ terminal of adjacent gene (Anderson et al. 1981). Gaojiao chicken was similar to other vertebrates, the lengths of 12s rRNA and16s rRNA were 976 and 1622 bp, which are located between the *tRNA^Leu^* and *tRNA^Phe^* genes and separated by the *tRNA^Val^* gene (Cheng et al. 2012).

The maximum-likelihood phylogenetic tree was constructed according to D-loop sequence of Gaojiao chicken and eleven chicken breeds from the National Center for Biotechnology Information (NCBI) (Figure 1). The results showed that the nucleotide sequences of the D-loop region of Gaojiao chicken were highly homologous with Nandan chicken, Daweishan chicken, Wuding chicken, and Guangxi three-buff chicken. We assumed that Gaojiao chicken has the closest relationship with Nandan chicken. However, Gaojiao chicken is farther distance with the Xiaoxiang chicken.

**Figure 1.**
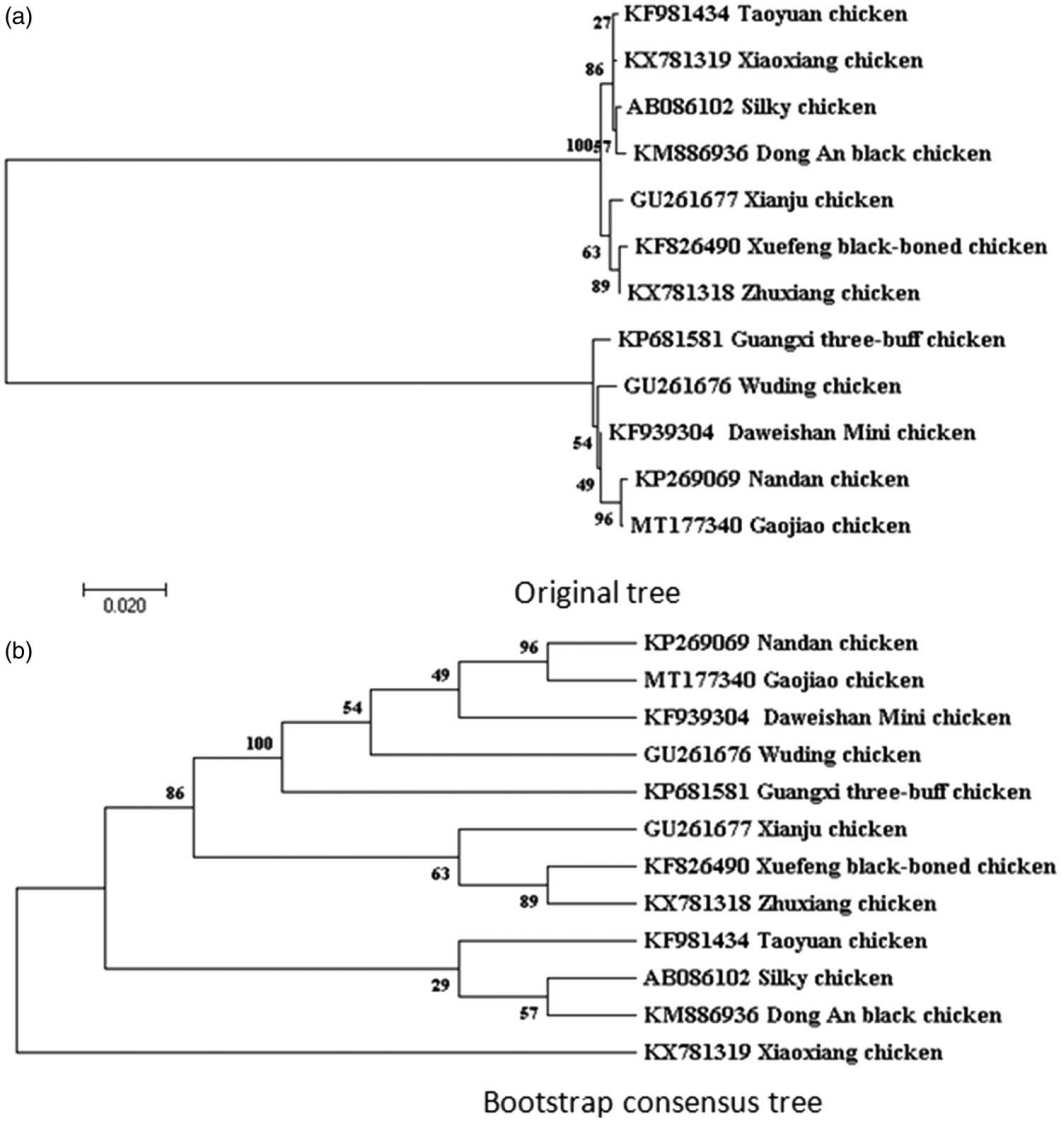
Based on D-loop sequence to construct phylogenetic tree; (a) Original tree and (b) bootstrap consensus tree in 12 chicken breeds. The mitochondrial DNA sequences of are downloaded from GenBank and the phylogenetic tree is constructed using a maximum-likelihood method on MEGA 7.0. The gene’s accession number for tree construction is listed as follow, Zhuxiang chicken (KX781318); Xiaoxiang chicken (KX781319); Taoyuan chicken (KF981434); Xuefeng black-boned chicken (KF826490); Dong An black chicken (KM886936); Wuding chicken (GU261676); Daweishan Mini chicken (KF939304); Guangxi three-buff chicken (KP681581); Xianju chicken (GU261677); Nandan chicken (KP269069); Silky chicken (AB086102).

## Data Availability

The data that support the findings of this study are openly available in GenBank at https://www.ncbi.nlm.nih.gov/genbank/, reference number MT177340.
